# Co-circulation of West Nile Virus Variants, Arizona, USA, 2010

**DOI:** 10.3201/eid2002.131008

**Published:** 2014-02

**Authors:** Jessica A. Plante, Kristen L. Burkhalter, Brian R. Mann, Marvin S. Godsey, John-Paul Mutebi, David W. C. Beasley

**Affiliations:** University of Texas Medical Branch, Galveston, Texas, USA (J.A. Plante, B.R. Mann, D.W.C. Beasley);; Centers for Disease Control and Prevention, Fort Collins, Colorado, USA. (K.L. Burkhalter, M.S. Godsey, Jr., J.P. Mutebi)

**Keywords:** West Nile virus, viruses, evolution, co-circulation, phylogenetics, variants, outbreak, Arizona, United States

## Abstract

Molecular analysis of West Nile virus (WNV) isolates obtained during a 2010 outbreak in Maricopa County, Arizona, USA, demonstrated co-circulation of 3 distinct genetic variants, including strains with novel envelope protein mutations. These results highlight the continuing evolution of WNV in North America and the current complexity of WNV dispersal and transmission.

West Nile virus (WNV) emerged in the Americas in 1999 after an outbreak of neuroinvasive disease in humans, birds, and horses in New York, New York. The virus spread rapidly across North America and was detected in Arizona in 2003. In 2004, Arizona experienced a large outbreak (214 neuroinvasive cases and 16 deaths, second only to California in that year), followed by ≈50–60 neuroinvasive cases per year during 2005–2008. 

An outbreak in 2010 resulted in 107 neuroinvasive cases and 15 deaths, the largest number of cases for a state that year. WNV activity in Maricopa County, which includes the city of Phoenix and surrounding municipalities, where numerous human cases were reported in the town of Gilbert, was investigated by a team from the Centers for Disease Control and Prevention (Fort Collins, CO, USA) working with local and state public health officials. Epidemiologic and entomologic findings from those investigations have been reported (*1*,*2*) We describe the molecular and phenotypic characterization of WNV isolates obtained from that outbreak.

## The Study

**Figure 1:**
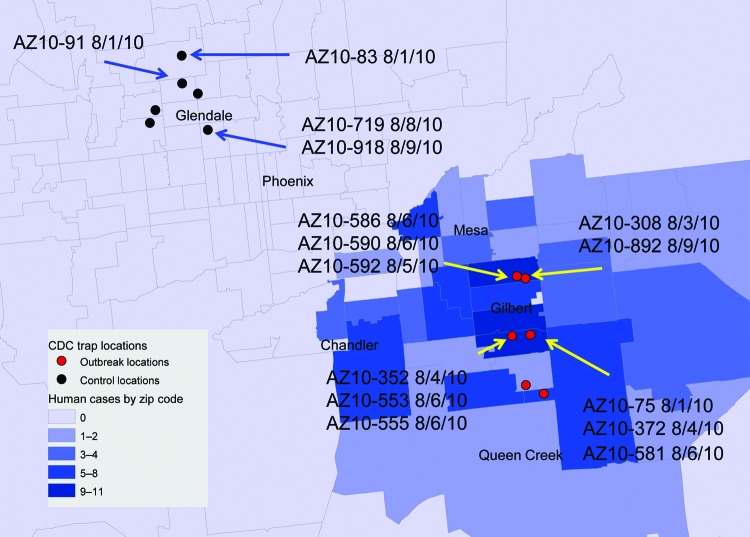
Distribution of mosquito sampling sites in Maricopa County, Arizona, USA, during the 2010 West Nile virus (WNV) outbreak investigation and collection dates/locations of pools yielding indicated WNV isolates used for molecular and/or phenotypic analysis. Gray lines indicate individual zip code boundaries. CDC, Centers for Disease Control and Prevention.

**Figure 2:**
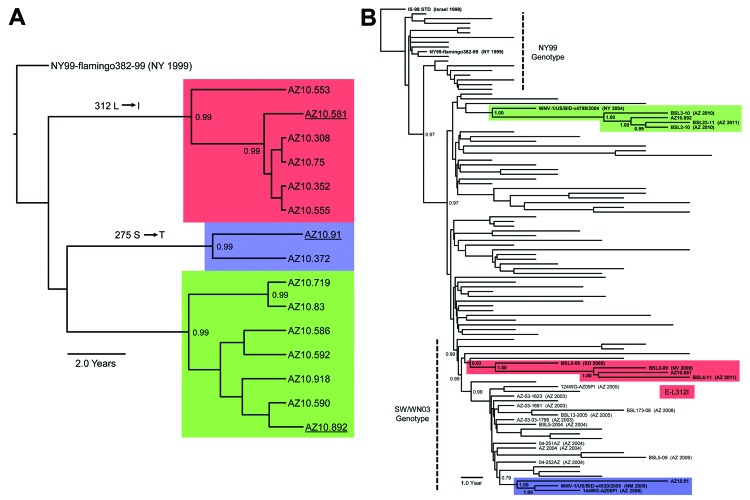
A) Bayesian phylogenetic tree of envelopes genes of all described Arizona, USA, 2010 isolates of West Nile virus (WNV) (n = 15). Isolates grouped in 3 distinct monophyletic clusters designated A (red), B (blue), and C (green). B) Bayesian phylogenetic tree of full-length encoded open reading frame for 3 Arizona, USA, 2010 isolates: AZ10.581 (red), AZ10.892 (green), AZ10.91 (blue), and 100 representative North American WNV isolates. All applied relaxed clock Bayesian methods used the generalized time reversible + invariant sites + Γ_4_ substitution model with a lognormal molecular clock and triplicate 50 million state runs produced in BEAST v1.6.2 (*3*). Inferred phylogenetic trees were edited in FigTree v1.3.1 (http://tree.bio.ed.ac.uk/software/figtree/). Consistent phylogenetic topologies with additional neighbor-joining and maximum-likelihood methods further validated these inferred relationships. Posterior probabilities ≥0.90 are indicated for highlighted nodes. Scale bars indicate divergence time in years.

As part of the Centers for Disease Control and Prevention investigation, Vero cell culture isolates of WNV were obtained from pools of *Culex quinquefasciatus* (Say) mosquitoes collected during August 1–9, 2010, at multiple sites in the study areas (Figure 1). Nucleotide sequences (GenBank accession nos. KF704145–KF704159) for the envelope (E) protein–coding regions were determined for 15 strains and subjected to phylogenetic analysis. The AZ10 E gene sequences were distributed in 3 robust monophyletic clusters (designated A, B, C; posterior probabilities 0.99) as determined by using applied relaxed clock Bayesian coalescent analysis (Figure 2, panel A).

Isolates from all 3 clusters were detected from the Gilbert study sites. All AZ10 isolates encoded the E-159 Val→Ala mutation that is characteristic of genotypes described since 2002 (*4*). The 7 strains in cluster A, which were only obtained from pools collected in Gilbert, also encoded a conservative Leu→Ile mutation at E-312, a surface exposed residue in the putative receptor binding domain III (EIII) known to be a variable site in multiple WNV genetic lineages, including strains of lineage 2 currently circulating in Europe (*5**–**9*). The 2 strains in cluster B, collected in Glendale and Gilbert, Arizona, encoded a conservative Ser→Thr mutation at E-275.

To further characterize the phylogenetic relationships of these AZ10 isolates, 1 strain from each cluster was selected for full-length genomic sequencing and comparison of the encoded open reading frames to 486 additional genomic sequences from North America available in GenBank. This analysis also supported the concurrent circulation of 3 distinct variants in Gilbert and the surrounding areas of Maricopa County during the 2010 outbreak (Figure 2, panel B). Strain AZ10–581 (cluster A) grouped with the recently described SW/WN03 genotype (*10*), and was most closely related to a South Dakota 2005 strain and 2 other strains that each encoded the E-L312I mutation. Strain AZ10–91 (cluster B) grouped with 2004–2005 Arizona and New Mexico isolates also belonging to a clade of the SW/WN03 genotype. Other SW/WN03 genotype viruses did not encode the E-275 mutation in AZ10–91 and AZ10–372. Strain AZ10–892 grouped with other recently described Arizona 2010/2011 isolates (*4*) and a New York 2004 strain belonging to the dominant NA/WN02 genotype, confirming persistence or reintroduction of that genotype in the southwestern United States (*4*). Nucleotide divergence from NY99 ranged from 0.58% to 0.66% for the AZ10 strains and divergence between the 3 clusters was up to ≈1.2% (Table 1).

**Table 1:** Nucleotide divergence for open reading frame sequences between representative West Nile virus strains and other closely related strains from North America, Arizona, USA, 2010*

Strain	Nucleotide divergence (%) from			
	NY99	Cluster A	Cluster B	Cluster C
AZ10–581	0.58	0.25–0.52	0.66–0.85	0.61–1.10
AZ10–91	0.66	0.74–1.04	0.49–0.52	0.69–1.16
AZ10–892	0.65	0.84–1.18	0.92–1.04	0.27–0.62

*Strains used for analysis in each cluster are those color coded with representative AZ10 strains and shown in Figure 2, panel B. For cluster A: BSL2–2005 (SD 2005; GenBank accession no. DQ666452), BSL2–09 (NV 2009, JF957175), BSL4–11 (AZ 2011, JQ700438); cluster B: v4530 (NM 2005, HM756677), 144WG-AZ06PI (AZ 2006, GQ507482); cluster C: v4798 (NY 2004, HM756671), BSL3–10 (AZ 2010, JF957186), BSL2–10 (AZ 2010 JF957185), BSL23–11 (AZ 2011, JQ700440).

The presence of the E-312 coding mutation was of particular interest. Most sequences for lineage 1 WNV strains encode Leu at E-312, whereas lineage 2 strains encode Val or Ala. E-312 lies in an exposed loop of EIII, where it may contribute to the antigenic and/or putative receptor binding activities of the domain (*6*). To assess the effects of the Leu→Ile mutation and tolerance for alternative amino acid substitutions at this site, we engineered 5 E-312 mutants by using an NY99 infectious clone (NY99ic) encoding alternative amino acids that are each only a single nucleotide substitution away from the wild-type Leu codon (CUU) (Table 2). (Although Phe also requires only a single nucleotide change from the Leu codon, it occurs naturally in some lineage 1 and 2 WNV strains and was not included in this analysis.) Mutagenesis, in vitro ligation, and transcription of genome equivalent RNA and virus recovery were performed as described (*11*). All mutant viruses were readily recovered from transfected Vero cells and grew to peak titers comparable to the parental NY99ic virus, and the introduced mutations were stable through 3 additional Vero cell passages.

**Table 2:** Mouse virulence and antigenic characteristics of selected isolates of West Nile virus and NY99ic-derived E-312 variants, Arizona, USA, 2010*

Strain/variant	Mouse neuroinvasiveness		Neutralization indices ± SD		
	ip LD_50_, PFU	AST ± SD (days)	7H2	5H10	α-EIII
NY99ic	0.3	8.7 ± 1.8	1.5 ± 0.2	1.5 ± 0.2	2.4 ± 0.3
AZ10–75	0.5	8.7 ± 2.2	1.3 ± 0.5	1.3 ± 0.3	2.1 ± 0.3
AZ10–581	0.8	9.8 ± 2.4	1.2 ± 0.2	1.3 ± 0.1	2.5 ± 0.2
AZ10–91	0.5	7.8 ± 0.9	ND	ND	ND
AZ10–892	0.3	8.6 ± 2.0	ND	ND	ND
NY99–312F	1.3	9.8 ± 1.9	1.5 ± 0.4	1.5 ± 0.4	2.5 ± 0.4
NY99–312H	0.8	8.5 ± 1.9	2.0 ± 0.1	1.7 ± 0.3	2.9 ± 0.0
NY99–312I	0.3	8.5 ± 1.4	1.8 ± 0.4	1.4 ± 0.3	2.5 ± 0.4
NY99–312P	630	**13.0** ± **0.0**	2.2 ± 0.1	2.0 ± 0.1	2.6 ± 0.2
NY99–312R	2.0	8.5 ± 2.2	1.8 ± 0.2	1.7 ± 0.1	2.7 ± 0.0

*Average survival time (AST) for each strain/variant was determined on the basis of animals in all dose groups that did not survive. Value significantly different (p<0.05 by Student *t*-test) from NY99ic is indicated in **boldface**. * ip, intraperitoneal; LD_50_, 50% lethal dose; ND, not determined.

Virulence of AZ10-75 and AZ10−581 (cluster A), AZ10-91 (cluster B), AZ10-892 (cluster C), and the recovered NY99ic E-312 mutants was compared with wild-type NY99ic after intraperitoneal inoculation of 3- to 4-week-old female Swiss Webster mice (Table 2) as described (*11*). The AZ10 strains and all E-312 mutants had 50% lethal doses (LD_50_s) and average survival times comparable with that of NY99ic, with the exception of the L312P mutant, which was markedly attenuated (630 PFU/LD_50_ vs. 0.3 PFU/LD_50_, and prolonged survival time). Antigenic characteristics of viruses encoding L312I mutations were also compared by assessing their neutralization by monoclonal antibodies 7H2 and 5H10 and a polyclonal rabbit antiserum against the EIII region as described (*5*). AZ10-75, AZ10-581 and all E-312 variants were effectively neutralized by the monoclonal antibodies and antiserum (Table 2).

## Conclusions

Detection of a Leu→Ile mutation at residue 312 in EIII and its apparent persistence since first detection in the 2005 South Dakota isolate were major findings given the variable nature of this residue in other WNV lineages and the presumed importance of EIII in antigenicity and receptor binding activity of E protein. Although other parameters that could contribute to the selection of E-312 variants in nature remain to be explored, analysis of engineered E-312 mutants suggested that most nonsynonymous single nucleotide mutations at this site, including the Leu→Ile substitution in some AZ10 isolates, have no major effect on virulence of NY99-derived WNV in mice and were not associated with major changes in antigenicity.

The 2010 epidemic of WNV disease in the Maricopa County area was associated with co-circulation of 3 distinct WNV variants. The high mouse virulence of all strains tested suggests that signature nucleotide and amino acid changes associated with the different genotypes involved (*4**,**10*) were probably not linked to major changes in virulence for mammalian hosts, and that all 3 variants might have contributed to human disease in the 2010 outbreak. Some nonstructural protein mutations have been shown to influence virulence in avian hosts (*12*), and that phenotype remains to be determined for these strains or other recently identified WNV variants.

Detection of multiple sequence variants has been associated with outbreaks in the United States in as early as 2002 (*13*), but co-circulation of variants in relatively narrow spatial and temporal contexts, such as that observed in Maricopa County, has been a feature of recent investigations, including WNV transmission in El Paso, Texas, and Ciudad Juarez, Mexico, during 2010 (*14*) and during the 2012 outbreak in Dallas, Texas (*15*). These findings highlight the current complexity and dynamic nature of WNV transmission in the United States and suggest that co-circulation of multiple variants, with continued introduction or reintroduction of variants into disease-endemic areas, will be a major feature of future outbreaks.
